# Leveraging Self-Reporting in an Existing e-Cohort to Identify Clinically Relevant Mitral Valve Prolapse: Pilot Questionnaire Study

**DOI:** 10.2196/77968

**Published:** 2026-06-24

**Authors:** Rohit Jhawar, Amy Rich, Luca Cristin, Lionel Tastet, Mark J Pletcher, Gregory M Marcus, Francesca N Delling

**Affiliations:** 1 Department of Medicine, Division of Cardiology, University of California San Francisco, 535 Mission Bay Boulevard South, San Francisco, CA, 94158, United States, 1 4155141162; 2 Department of Epidemiology and Biostatistics, University of California, San Francisco, San Francisco, United States

**Keywords:** valvular heart disease, digital health, e-cohort, recruitment, arrhythmias

## Abstract

**Background:**

Mitral valve prolapse (MVP) is a common valvulopathy associated, in a minority of cases, with heart failure, severe mitral regurgitation (MR), and sudden arrhythmic death. Digital tools hold promise for faster and more efficient recruitment of study participants into a large-scale MVP Registry.

**Objective:**

This study sought to evaluate the feasibility of surveying participants in an existing e-cohort to identify and clinically characterize MVP cases based on self-reporting and to recruit them in an MVP Registry at the University of California, San Francisco.

**Methods:**

We surveyed Northern Californian participants of the Health eHeart Study, a large e-cohort using the Eureka digital research infrastructure, about a prior diagnosis of MVP. MVP-positive respondents were asked to provide relevant medical records to confirm their eligibility and were invited to enroll in an MVP Registry if evidence of MVP was confirmed. A follow-up survey was sent after 1 month and after 5 years to collect data about clinical outcomes, including arrhythmias and the need for mitral valve repair.

**Results:**

The survey was delivered to 5746 participants, and 520 completed responses were collected. A prior diagnosis of MVP was self-reported by 16.3% (85/520) of respondents. Echocardiograms were obtained from 51.8% (44/85) of participants, and evidence of MVP was confirmed in 32.9% (n=28) of individuals, all of whom joined the registry. Participants with more severe MR had a higher number of correct responses regarding both MVP (odds ratio [OR] 10.58, 95% CI 3.58‐63.04; *P*<.001) and MR diagnosis (OR 4.86, 95% CI 2.11‐16.14; *P*=.002). Longitudinal data were available from most patients through responses to a follow-up survey sent 1 month and 5 years later (18/28, 64.3% and 17/28, 60.7% of MVP confirmed respondents, respectively). Among the patients with electronic health records available, 75% (3/4) had a correct self-reported diagnosis of arrhythmia.

**Conclusions:**

e-Cohort methods with self-reported clinical data can be used to prescreen candidates for a research study of MVP. These methods can rapidly identify and retain, among many cases of benign MVP, the minority with clinically relevant presentations such as significant MR and ventricular arrhythmias. These cases may be missed, especially when asymptomatic, by small-scale clinic-based recruitment or family screening methods.

## Introduction

**Figure 1.**
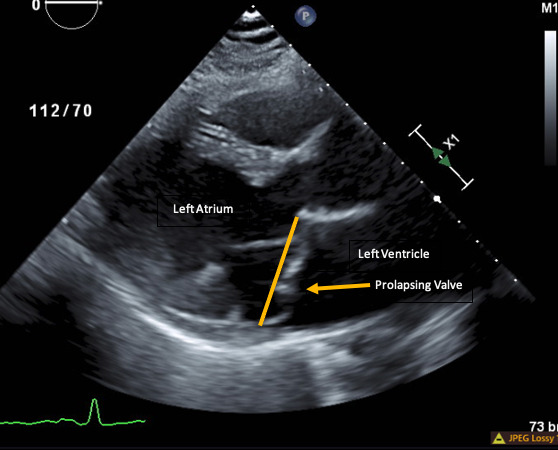
Echocardiographic parasternal long-axis view demonstrating mitral valve prolapse, characterized by systolic displacement of the mitral valve leaflets ≥2 mm beyond the plane of the mitral annulus.

Mitral valve prolapse (MVP) is a common valvular disorder that affects about 2% to 3% of the general population [1,2]. MVP is characterized by leaflet thickening and is defined by echocardiography as leaflet displacement >2 mm beyond the mitral annulus in a long-axis view at end-systole (Figure 1) [3]. MVP is often a benign finding, but a minority of individuals may develop complications, including severe mitral regurgitation (MR), heart failure, and even sudden cardiac death (SCD) [4]—a devastating outcome that can affect younger, asymptomatic individuals with MVP. Indeed, analyses of SCDs in young adults with MVP have shown that a significant number of patients had no previous cardiology evaluations [5]. Identifying individuals at increased risk among the large population of patients with otherwise uncomplicated MVP may help reduce morbidity and mortality.

Patient registries are invaluable tools for studying patients who share a particular condition, treatment history, or other characteristics. Registries allow for a centralized data collection process across multiple individual studies and enable rapid and cost-efficient investigation of new questions. Registries are particularly helpful for studying rare and complex phenotypes such as MVP with SCD. We have recently developed a University of California, San Francisco (UCSF)–based registry of 807 patients with an echocardiographic diagnosis of MVP between 2017 and 2024. The registry includes clinical, electrocardiographic, and cardiac imaging data. The maintenance of a diverse registry has enabled us to efficiently investigate a number of questions regarding predictors of adverse outcomes in MVP, including overall death, SCD, ventricular arrhythmias, and atrial fibrillation [6-10]. Our findings have prompted interest in strategies to improve efficiency as we continue to expand and update the registry.

The main challenges facing patient registries, including our own, are recruiting a broad and representative patient population and ensuring the collection and continuous updating of clinical information. Although registries vastly decrease the individual burden of downstream studies, they require a significant investment in time, effort, and resources spent up front. This investment is mainly related to the screening of potential participants and communication during enrollment.

Digital methods offer powerful solutions to these challenges and are promising tools for establishing, maintaining, and growing patient registries. The broad reach of technology may simplify recruitment from larger populations and potentially enable enrollment of more diverse samples. Digital tools could streamline patient enrollment and communication and simplify the process of recruiting and collecting data from many patients in parallel rather than in series (eg, surveys can be emailed to hundreds of patients at the same time rather than administered in person or over the phone one by one). Many existing patient registries have recently sought to leverage these advantages, and they have shown promising preliminary results [11-14].

The Health eHeart (HeH) Study is an internet-based, longitudinal cardiovascular cohort study of adults that currently has more than 250,000 consented individuals enrolled from around the globe. This includes representation from every state in the United States. The HeH platform is designed for mobile research, broadly defined as any research using mobile apps, sensors, connected devices, and/or the internet. The primary strength of the HeH Study is a UCSF Institutional Review Board–approved internet-based remote consent. This infrastructure obviates the cost of in-person coordinators and can support the consent of tens of thousands of individuals regardless of their physical location or place of residence. Recently, the HeH was incorporated into the larger Eureka digital research infrastructure [13]. Eureka is a comprehensive system for direct-to-participant data collection and study management available for use across major consumer operating systems, including iOS and Android smartphones.

Our primary objective was to test the feasibility of using a digital survey to recruit participants with MVP into our registry based on a self-reported diagnosis of MVP. We then sought to test the feasibility of using follow-up digital communication methods to collect clinical data from these individuals and track long-term outcomes.

## Methods

### Study Population

Enrollment, consent, and participation in the HeH Study were fully digital, with recruitment of participants occurring via news stories, social media, and email campaigns, and referrals or word of mouth, as previously described [13,15]. Participation in the HeH Study was open to adults (aged ≥18 y) who understand English and have an active email. HeH participants completed a series of questionnaires upon enrollment, mainly pertaining to demographics, lifestyle, and basic clinical information. We identified 5746 HeH participants living in Northern California (based on zip code) and sent an MVP-focused questionnaire via email to this group.

### Baseline Survey Design

The email invitation to complete the MVP-focused questionnaire mentioned “Mitral Valve Prolapse Survey” in the subject line. Questions pertained to MVP diagnosis, history of echocardiograms, MR diagnosis, and prior surgical recommendations. The questionnaire is provided in Table S1 in Multimedia Appendix 1. The email invitations were sent in 3 waves, each sent on a separate day about 2 weeks apart, between December 2016 and January 2017. Patients were able to leave questions blank, and responses were collected up to 1.5 years after the survey was sent. A complete survey was defined as one in which no questions were left blank.

### Recruitment

All survey responses were reviewed, and individuals who self-reported an MVP diagnosis were contacted by either phone call or email to confirm MVP through the availability of an echocardiographic report. If the participants were UCSF patients, echocardiographic reports were obtained directly from the UCSF electronic health record. Report authenticity was confirmed by matching patient names and dates of birth to the headers on the report. After a report was reviewed and the MVP diagnosis was confirmed, participants were contacted by either phone call or email and asked to schedule a longer phone call. This longer call involved a description of our MVP Registry and the type of data collected, followed by an inquiry regarding the participant’s interest in enrolling. If interested, they were sent an email containing a DocuSign (Docusign, Inc) link to sign the consent form and formally join our MVP Registry.

### Follow-Up Survey

All individuals successfully recruited to the study were emailed 2 follow-up surveys: the first 1 month after the initial survey (Table S2 in Multimedia Appendix 1) and the second 5 years later (Figure S3 in Multimedia Appendix 1). The first follow-up was conducted through the HeH infrastructure, while the second survey was directly emailed to patients and administered through Research Electronic Data Capture (REDCap; Vanderbilt University). Both surveys were designed to collect clinical data from patients, with a focus on long-term outcomes in the 5-year follow-up.

### Clinical Data Verification

Responses to baseline survey questions were compared to echocardiographic findings for participants who provided an echocardiographic report. The appearance of MVP on echocardiography is shown in Figure 1. On echocardiography, the severity of MR was determined using a multiparametric approach, as recommended by current guidelines [16]. Responses to clinical history questions were validated with electronic health record data for patients who had previously received care at UCSF Cardiology. Consent for viewing electronic health record data was included in the overall MVP Registry consent. Questions were chosen for validation based on the presence and accessibility of a source of ground truth for corroboration.

### Outcomes

The primary outcomes were (1) validation of a self-reported diagnosis of MVP (with clinically relevant MVP defined as MVP with moderate or greater MR or any ventricular arrhythmia) in an e-cohort through review of echocardiographic reports and electronic health records and (2) the number of individuals with MVP who could be successfully enrolled in our MVP Registry. Secondary outcomes of interest were retention rate in follow-up communications, demographic diversity of recruited patients, and the comprehensiveness of collected clinical characteristics and outcomes.

### Statistical Analysis

Continuous, nonnormally distributed variables are presented as medians with IQRs and were compared using the Kruskal-Wallis test. Categorical variables were compared using the Mann-Whitney *U* test. Binary variables were compared using contingency tables and Fisher exact test. All logistic regressions were univariate analyses. Sensitivity was calculated as true positives divided by the sum of true positives and false negatives. Specificity was calculated as true negatives divided by the sum of true negatives and false positives. A 2-tailed *P*<.05 was considered statistically significant. All analyses were conducted in RStudio (version 4.5.0; R Foundation for Statistical Computing; installed packages: *ggplot2*, *flowchart*, *tidyverse*, and *dplyr*).

### Ethical Considerations

This study was approved by the UCSF Institutional Review Board (study ID 17-22679). Informed consent was obtained before participants enrolled in the HeH platform and before enrollment in the MVP Registry. All data were anonymized. No compensation was provided for survey completion.

## Results

### Survey Response Rate

**Figure 2.**
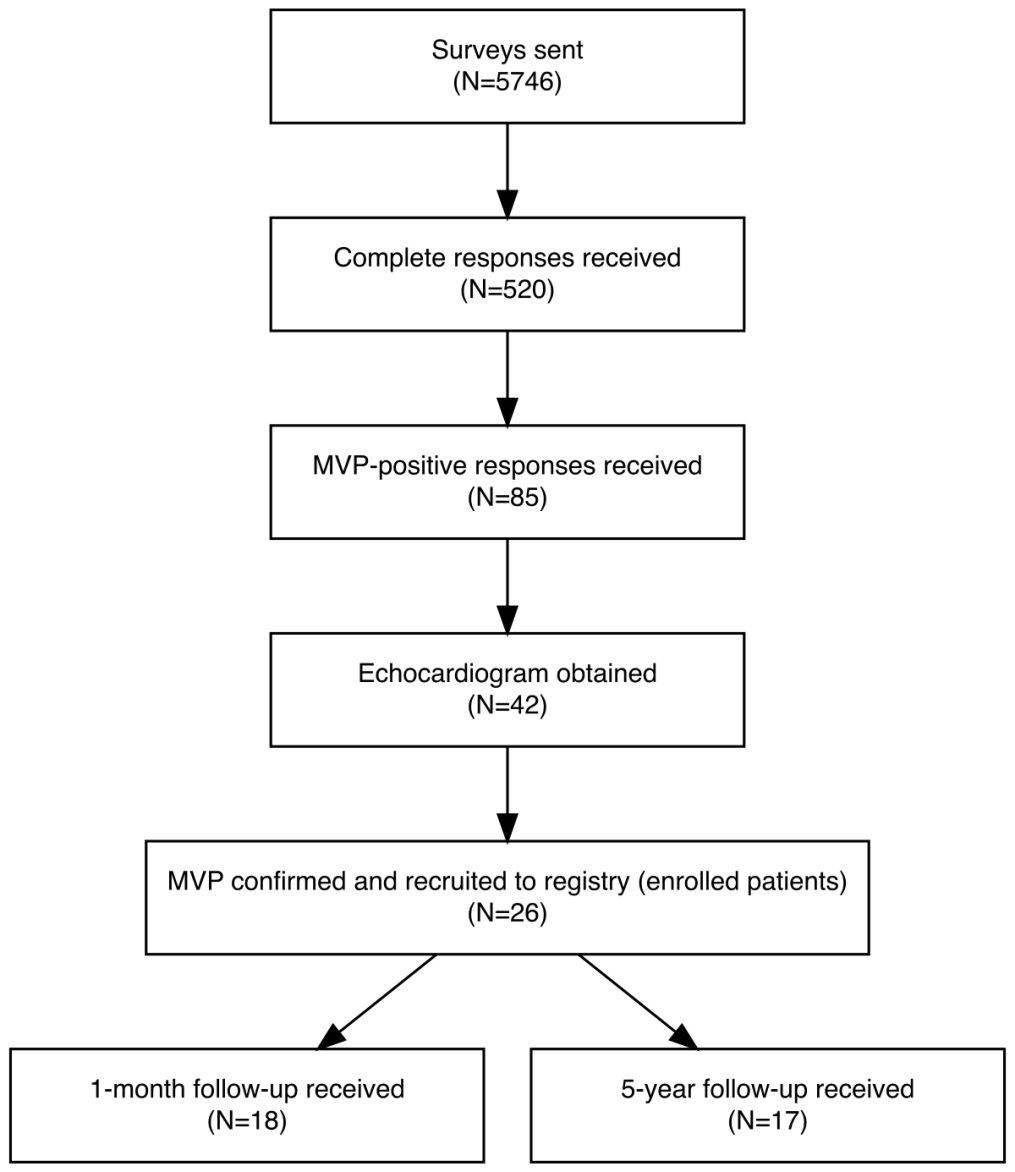
Flowchart indicating the retention of study participants across different stages of the survey. MVP: mitral valve prolapse.

Out of the 5746 participants who received the survey, 520 (9%) submitted complete responses. In total, 16.3% of the participants self-reported an MVP diagnosis (MVP-positive respondents). The flowchart is displayed in Figure 2.

### Respondent Demographics

The median age of MVP-positive respondents was 66 (59-72) years, and 65.9% (56/85) of the MVP-positive respondents self-identified as female (Table 1). A total of 66 zip codes and 44 cities were represented in this pool. In total, 10.6% (n=9) of the MVP-positive respondents were born outside of the United States, and 68.2% (n=58) of the MVP-positive respondents were not San Francisco residents. Out of the 85 MVP-positive respondents, 62 (72.9%) were White, 20 (23.5%) were other or unknown, 2 (2.4%) were Asian, and 1 (1.2%) was Black. Notably, 55.3% (n=47) of the MVP-positive respondents were not UCSF Cardiology patients.

**Table 1.** Baseline characteristics of mitral valve prolapse–positive respondents.

	Baseline (n=85)	No follow-up (n=62)	1-month follow-up (n=18)	5-year follow-up (n=17)	*P* value^a^
Age at baseline (y), median (IQR)	66 (59-72)	66 (59-72)	70 (60-76)	63 (56-72)	.91
Female, n (%)	56 (66)	45 (73)	9 (50)	8 (47)	.08
Race, n (%)					.27
Asian	2 (2)	1 (2)	1 (6)	1 (6)	
Black	1 (1)	1 (2)	0 (0)	0 (0)	
Other or unknown	20 (24)	18 (29)	1 (6)	2 (12)	
White	62 (73)	42 (68)	16 (89)	14 (82)	
Born outside of the United States, n (%)	9 (11)	7 (11)	1 (5)	2 (12)	.99
Not San Francisco residents, n (%)	58 (68)	42 (68)	13 (72)	12 (71)	.99

a *P* value for the comparison between no follow-up and 5-year follow-up. Patients who completed the 1-month follow-up may also be in the 5-year follow-up category if they completed both.

### Self-Reported MVP and Clinical Correlates

In total, 94.1% (80/85) of the MVP-positive respondents indicated that their diagnosis had previously been confirmed by echocardiography. Moreover, 57.6% (n=49) of the MVP-positive respondents reported a “leaky valve” as a consequence of their prolapse, and 22.4% (n=19) of the MVP-positive respondents reported being told they needed mitral valve surgery.

### Diagnosis Verification and Recruitment

Among the 85 MVP-positive respondents, 54 (63.5%) indicated interest in participating in the MVP Registry. Echocardiogram reports were sent by 8 participants directly via email and were obtained for 36 others through UCSF Cardiology (44 total echocardiograms obtained). After reviewing the available echocardiograms, evidence of MVP was present on 59.1% (26/44) of the echocardiograms, and prior mitral valve repair was present on 4.5% (n=2) of the echocardiograms (Table 2). Correct self-reporting of MR history (both yes and no) occurred in 32 (73%) out of the 44 echocardiograms. Of the 44 individuals who submitted echocardiograms, 16 (36.4%) self-reported a diagnosis of MVP that was not confirmed by echocardiography. The majority (12/16, 75%) of these “false positive” respondents were older than 60 years.

**Table 2.** Study participants with available echocardiogram, stratified by evidence of mitral valve prolapse.

	No evidence of prolapse (n=16)	Evidence of prolapse (n=28)	*P* value
Age (y), median (IQR)	64 (54-68)	71 (62-74)	.051
Female, n (%)	11 (69)	14 (50)	.34
Severity of mitral regurgitation on echocardiogram, n (%)			<.001
None	12 (75)	0 (0)	
Mild	2 (13)	7 (25)	
Moderate	2 (13)	6 (21)	
Severe or prior valvular intervention	0 (0)	15 (54)	
Correctly reported mitral regurgitation status on baseline survey, n (%)	8 (50)	24 (86)	.02
Need for surgery, n (%)	2 (13)	15 (54)	.008

**Figure 3.**
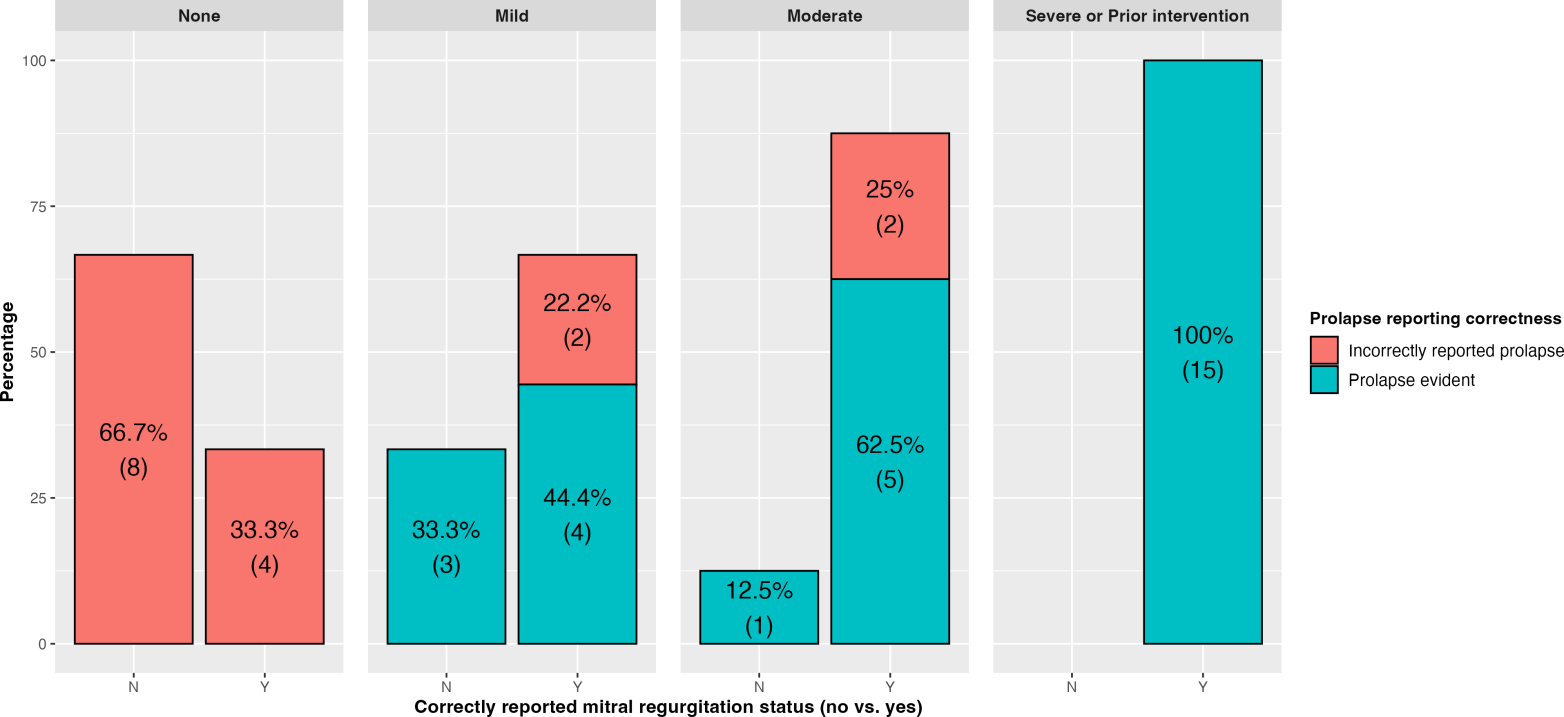
Accuracy of reporting mitral valve prolapse and mitral regurgitation (MR), stratified by MR severity on baseline echocardiogram. Each panel represents a different grade of MR and is split into 2 bars indicating correct MR reporting (blue) and incorrect MR reporting (red).

The sensitivity of MR reporting was 88% (95% CI 0.71‐0.96) and specificity was 33% (95% CI 0.09‐0.65). Participants who correctly reported MVP were significantly more likely to have a correctly reported MR status (24/28, 86% vs 8/16, 50%; *P*=.02). In univariate logistic regression, greater MR severity was significantly associated with having a correct, echocardiographically confirmed diagnosis of MVP (odds ratio [OR] 10.58, 95% CI 3.58‐63.04; *P*<.001) and MR (OR 4.86, 95% CI 2.11‐16.14; *P*=.002). These results are visualized in Figure 3, stratified by MR severity. All participants with trace or physiologic MR erroneously reported MVP, and participants with severe regurgitation or a prior intervention were correct about both their MVP and MR history. Participants who had been told they needed surgery were also significantly more likely to have correctly reported MVP (15/28, 54% vs 2/16, 13%; *P*=.008). All 28 participants with confirmed MVP signed consent forms and enrolled in the MVP Registry.

### 1-Month Follow-Up Survey

Of the 28 participants with confirmed MVP, 18 (64.3%) responded to the follow-up survey sent 1 month later. Demographics are displayed in Table 1: there were no significant differences between respondents and nonrespondents at follow-up; 14 (78%) out of the 18 respondents were female, and the median age was 70 (IQR 60-76) years; 61.1% (n=11) of the respondents indicated a history of palpitations, with 7 (64%) of these 11 respondents having seen a medical provider about these symptoms; of those who had been evaluated by a physician regarding palpitations, 57.1% (4/7) reported ventricular ectopy and 28.6% (n=2) reported atrial fibrillation as the etiology of the palpitations. In total, 22.2% (4/18) of the respondents reported a prior syncopal episode, and 33.3% (n=6) reported a family history of cardiac arrest; 5.6% (n=1) of the respondents reported a history of ventricular ablation and implantation of an implantable cardioverter-defibrillator following a cardiac arrest.

### 5-Year Follow-Up Survey

Of the 28 participants with confirmed MVP, 17 (60.7%) responded to the follow-up survey sent 5 years later. Demographics are shown in Table 1: there were no significant differences between respondents and nonrespondents at 5-year follow-up. The median age was 63 (IQR 56-72) years, and 47% (n=8) of the respondents were female. The results of the survey are shown in Table 3, stratified by whether the patient had severe MR or a prior intervention at baseline, and include type of arrhythmia, symptoms, and history of mitral valve surgery. Premature ventricular contractions (PVCs) were reported by 11.7% (n=2) of the respondents, ventricular fibrillation or cardiac arrest by 6% (n=1) of the respondents, and ventricular tachycardia (VT) by 6% (n=1) of the respondents. Shortness of breath was reported by 64.7% (n=11) of the respondents, dizziness or lightheadedness by 35.2% (n=6) of the respondents, syncope by 11.7% (n=2) of the respondents, chest pain by 11.7% (n=2) of the respondents, and palpitations by 52.9% (n=9) of the respondents. The history of mitral valve surgery was reported by 47% (n=8) of the respondents.

**Table 3.** Results of the 5-year follow-up survey, stratified by presence of severe mitral regurgitation (MR) or prior intervention at baseline.

	No severe MR or prior intervention at baseline (n=7)	Severe MR or prior intervention at baseline (n=10)
Age (y), median (IQR)	59 (55-68)	70 (61-73)
Female, n (%)	5 (71)	3 (30)
Arrhythmias, n (%)		
Premature ventricular contractions	1 (14)	1 (10)
Ventricular fibrillation or cardiac arrest	0 (0)	1 (10)
Ventricular tachycardia	0 (0)	2 (20)
Unsure which arrhythmia	0 (0)	1 (10)
Symptoms, n (%)		
Shortness of breath	4 (57)	8 (80)
Presyncope	4 (57)	3 (30)
Syncope	2 (29)	1 (10)
Chest pain	2 (29)	1 (10)
Palpitations	5 (72)	5 (50)
Weakness	0 (0)	1 (10)
Mitral valve surgery, n (%)	1 (14)	9 (90)
Initial trigger of MVP^a^ diagnosis, n (%)		
Physical examination or routine imaging	4 (57)	7 (70)
Incidental discovery	1 (14)	2 (20)
Symptomatic	4 (57)	1 (10)
Family history	0 (0)	0 (0)
Method used for initial identification of MVP, n (%)		
Auscultation	6 (86)	6 (60)
Imaging	6 (86)	9 (90)
Surgical evidence	1 (14)	7 (70)

a MVP: mitral valve prolapse.

### Method of MVP Diagnosis

Out of the 17 respondents, 11 (64.7%) indicated that their initial MVP diagnosis was based on routine physical examination or imaging, 3 (17.6%) indicated that MVP was incidentally discovered during evaluation for other conditions, and 5 (29.4%) indicated that MVP was discovered during evaluation of symptoms; no respondents reported undergoing evaluation for MVP due to a family history of the condition. Despite MVP diagnosis having been previously confirmed by echocardiography for all respondents, only 88.2% (n=15) of the respondents reported that their MVP diagnosis had been confirmed by imaging, 70.6% (n=12) reported confirmation by prior auscultation, and 47.1% (n=8) reported confirmation by surgical intervention.

### Electronic Health Record Validation

Among the 17 respondents who responded to the 5-year follow-up survey, 14 (82.4%) had previously been seen by, or were currently under the care of, a UCSF cardiologist. Thus, their electronic health record was available for verification of their self-reported symptoms and treatment of MVP. All patients who indicated a history of prior MV surgery had evidence of a prior intervention. Participants who reported arrhythmias did have evidence of these in 75% (3/4) of the cases, with most reported arrhythmias being of clinical relevance after review of electronic health records: 2 had PVCs and 1 had VT. Only 1 participant was found to have incorrectly positively affirmed a condition: this individual had self-reported a prior ventricular fibrillation or cardiac arrest episode, but the medical record indicated no prior history of ventricular arrhythmias or syncopal episodes. This patient did have atrial fibrillation. Negative affirmation errors were common: 8 participants had a history of PVCs they did not self-report and 6 had a history of VT that was not self-reported. Atrial fibrillation was present in 55.6% (5/9) of the participants with negative affirmation errors, and 66.7% (n=6) of the participants with negative affirmation errors were asymptomatic.

## Discussion

### Principal Findings

In this study, we demonstrate the feasibility of surveying participants in an existing e-cohort using a fully digital mobile tool that allows self-reporting of MVP and clinical outcomes. Compared to small-scale in-person recruitment during a clinic visit, the HeH digital tool will facilitate large-scale and out-of-state recruitment of individuals into our UCSF MVP Registry. We also report the demographic and clinical diversity of recruited individuals with MVP, as well as retention up to 5 years after enrollment. Participants with clinically relevant MVP phenotypes (significant MR and/or ventricular arrhythmias) were more likely to accurately report their medical history. Moreover, participants did not make positive affirmation errors when asked about clinical sequelae. Overall, we found that e-cohort recruitment methods are valuable for prescreening individuals who might qualify for our MVP Registry and for streamlining future communications with them.

### Benefits of a Self-Reported Diagnosis of MVP in an Existing e-Cohort

The clearest potential value of electronic clinical research platforms comes from the ability to rapidly engage a large group of participants across different states and types of medical facilities. It is well established that tertiary care clinic–based recruitment and other traditional methods are often limited by selection bias, as research participants tend to also be the most symptomatic patients in need of medical attention. However, our study demonstrates the relative ease of digital methods as a supplemental tool. Our protocol enabled rapid, low-cost, and unbiased screening of thousands of individuals with self-reported MVP to identify study candidates. Our results revealed that such methods yield a wide spectrum of study participants with varying clinical severities. Notably, we maintained near-gender parity throughout the entire context of candidate identification, recruitment, and follow-up, indicating a significant advantage of digital methods over traditional recruitment. We were also able to engage a substantial number of non-San Francisco residents throughout the entire course of the study, further suggesting that we were accessing a study population that could be more difficult to engage with in-person methods. Given such improvements, and as digital tools continue to become more universally available and used, we anticipate that such protocols will be able to access larger and more diverse patient populations in the future and will enable an even more streamlined process for patient communication.

### Validation of Self-Reported MVP and Recruitment Into an MVP Registry

Most electronic infrastructures focus on enabling a simple workflow to communicate with patients and bidirectionally share information with them. Our protocol leveraged this workflow and saved time by ensuring study that staff only call patients who have already expressed initial interest. However, prior analyses of the veracity of self-reported clinical information have revealed meaningful concerns about the reliability of these data, especially in the context of affirming positive histories of a condition. Although self-reporting of MVP was validated through review of echocardiographic reports in 64% (28/44) of the cases, there were 36.3% (n=16) individuals, mostly older than 60 years, who self-reported MVP without evidence of MVP on echocardiography. This may be explained by the history of MVP diagnosis. Throughout most of the 20th century [3,17-19], MVP was diagnosed by relatively error-prone cardiac auscultation and by the observance of prolapse in any standard echocardiographic view. The prevalence of MVP was estimated to be up to 33%, until suspicion of overdiagnosis led researchers in the 1980s to discover that some echocardiographic views show MVP as a geometric artifact even when patients do not have it [3]. The guidelines were revised in 1989 to clarify that only the parasternal long-axis view, which transects the annular saddle at its highest points, was appropriate for diagnosing MVP, and the prevalence dramatically decreased to 2% to 5% [1]. However, many of the patients who were diagnosed with MVP prior to the widespread adoption of these revised criteria may still erroneously believe that they have the condition today. Cases erroneously diagnosed with MVP may not have undergone repeat echocardiography because MR was trivial and the treating physician may not have considered serial imaging necessary in the absence of symptoms. Moreover, if individuals with a prior erroneous diagnosis of MVP were later reevaluated and found not to have MVP on echocardiography, they may not be aware of this change in diagnostic status. Overall, high sensitivity of MVP diagnosis is preferred over low specificity: the ideal screening tool should be able to adequately detect true disease (high true positive rate) among all ages, whereas low specificity of self-reporting in our study was an issue pertaining mainly to older individuals (erroneously told they had MVP prior to the 1989 revised MVP diagnostic criteria). Therefore, older individuals may require confirmation of echocardiographic diagnosis, whereas younger study participants may not.

Interestingly, we found much lower positive affirmation inaccuracy rates for clinical sequelae of MVP, such as the need for surgical intervention and ventricular arrhythmias. Our results regarding MR severity and history of surgical intervention suggest that patients with more severe phenotypes may be more reliable historians. Conversely, patients with more benign or asymptomatic phenotypes were far more commonly incorrect about their diagnoses. Hence, self-reporting can generally be relied on with regard to the presence of clinical sequelae for confirmed MVP but should be externally validated to confirm the absence of sequelae.

### Follow-Up Rates and Richness of Clinical Data

In-person study methods can often have large amounts of study participants lost to follow-up, with the potential for bias due to the differential characteristics of patients who follow-up. Our study revealed a powerful advantage of digital methods in the relative similarity between demographics of participants in the baseline group who did not follow-up and those who remained in the study after 5 years. We observed that above half of the participants who agreed to join the study responded to the 5-year follow-up, indicating a satisfactory retention rate after enrollment. The 5-year follow-up survey also indicated that no study participants had MVP diagnosed due to family screening methods, suggesting that family screening–based recruitment is not commonly used in clinical practice.

The obvious advantage of in-person methods over digital ones is the depth and granularity of clinical data that can be collected in person. Our study did find a meaningful amount of negative affirmation errors when participants were asked about specific arrhythmia histories, suggesting that our protocol was insufficient for overcoming this limitation of digital methods. Further studies are necessary to verify whether increasing depth of questions is correlated with decreases in accuracy, and then to design protocols that might increase reliability when asking granular questions. Still, for questions where self-reporting is the existing gold standard (eg, symptoms), this protocol had satisfactory response rates and may suffice.

### Limitations

A traditional limitation pertaining to digital methods is the potential bias toward younger participants who are more avid users of digital tools. However, we did not observe this in our study, as we saw a fairly wide age range of participants throughout the entire course of the study. A major limitation of this protocol was the low response rate to the initial survey. This could be addressed in future protocols with measures such as follow-up reminders, offering assistance for participants who need help completing it, and incentives for participation.

Moreover, there was an approximately 50% drop-off between candidates who may have qualified based on the baseline survey and those who provided an echocardiogram for verification. Two plausible explanations for this attrition are the limited infrastructure within electronic health records for patients to easily obtain copies of their records and the similarly limited functionality within the HeH app for uploading such records. Both issues have significantly changed since the initial administration of this survey, as patient-side interfaces of electronic health records have become more expansive and the HeH Study has moved to the robust and comprehensive Eureka digital infrastructure. Although there were no deaths based on UCSF electronic health records, additional information about deaths outside of UCSF through the National Death Index was not available. Therefore, we cannot exclude competing risk of mortality as a possible reason for lack of survey responses or study attrition. Finally, surveys were entirely in English, which may have limited completion by non-English speakers.

### Conclusions

Our study demonstrates that self-reporting of MVP and its clinical sequelae can be validated in a large e-cohort. Hence, a digital infrastructure can be used as a supplementary tool for recruiting patients in an MVP Registry. On the basis of metrics such as demographic diversity, information accuracy, and study retention rate, we foresee use of this digital tool for prescreening study participants within a US-wide population that we might not otherwise be able to engage and for tracking retention in the study in the long term.

## Supplementary material

10.2196/77968 Multimedia Appendix 1 Supplementary tables showing survey design.

## Abbreviations

HeH: Health eHeart
MR: mitral regurgitation
MVP: mitral valve prolapse
OR: odds ratio
PVC: premature ventricular contraction
REDCap: Research Electronic Data Capture
SCD: sudden cardiac death
UCSF: University of California, San Francisco
VT: ventricular tachycardia
