# Supplementary material for: Leveraging Self-Reporting in an Existing e-Cohort to Identify Clinically Relevant Mitral Valve Prolapse: Pilot Questionnaire Study
Source: JMIR Form Res. 2026 Jun 24;10:e77968. doi: 10.2196/77968 (PMC13293562; doi:10.2196/77968)

**Supplemental Figure 1:** Initial questionnaire sent to all Northern California Health eHeart participants through the Eureka platform.

| Question?  ***Popup Language Title***  ***Popup Language Body***  ***variable name***  ***field type***  ***branching logic***  ***required/not required*** | 1, Option #1  2, Option #2  **Text Validation**  **Triggering Between Survey**  ***Note - questions that are triggered have a blue “fill”*** |
| --- | --- |
|  |  |
| Do you have mitral valve prolapse?  ***mvp***  ***radio***  ***none***  ***required***  *****this question will only be included in surveys sent via email to participants*** | 1, Yes  2, No  3, I don’t know |
| Has your diagnosis of mitral valve prolapse been confirmed with an ultrasound (also called an “echocardiogram” or “echo”) of your heart?  ***This involves applying gel to your chest and using a probe to obtain moving images of your heart.***  ***mvp_echo***  ***radio***  ***[mvp] = ‘1’***  ***required*** | 1, Yes  2, No  3, I don’t know |
| Where was that ultrasound (or echocardiogram) performed?  ***echo_place***  ***string***  ***[mvp_echo] = ‘1’***  ***required*** | Text box |
|  |  |
| Please provide your best approximation of the date of that study (if you do not remember, please make your best guess.  ***echo_date***  ***string***  ***[mvp_echo] = ‘1’***  ***required*** | Date text box |
|  |  |
| Have you been told that you have a leaky valve as a consequence of mitral valve prolapse?  ***leaky_valve***  ***radio***  ***none***  ***required*** | 1, Yes  2, No  3, I don’t know |
|  |  |
| Have you been told that you need surgery for the leaky valve?  ***surg_lv***  ***radio***  ***none***  ***required*** | 1, Yes  2, No  3, I don’t know |
|  |  |

Supplemental Figure 2: One month follow up survey sent to all enrolled MVP positive respondents through the Eureka platform.

| 1-09 Symptoms Overview Survey | |
| --- | --- |
| Do you ever get:   - palpitations - fast heart beats or racing heart rate (not due to normal exercise) - irregular heart beats - skipped beats, or - any funny feelings of abnormal heart beats?   ***palpitations***  ***yesno***  ***none***  ***required*** | 1, Yes  0, No  **[palpitations] = ‘1’, triggers:**   - **1-9c Palpitation Symptoms** |
| Have you ever fainted or passed out (lost consciousness) or been told you have syncope (not due to seizures, low blood sugar or an accident or other trauma)?  ***syncope***  ***yesno***  ***none***  ***required*** | 1, Yes  0, No |
| 1-09c Palpitations Survey | |
| Have you seen a doctor or nurse about this (include being hospitalized or a visit to an emergency room for this)?  ***seen_doctor***  ***dropdown***  ***none***  ***required*** | 1, Yes  0, No |
| Have you been told the cause by a doctor or nurse?  ***told_cause***  ***dropdown***  ***none***  ***required*** | 1, Yes  0, No |
| What is the cause? Check all that apply.  ***cause***  ***checkbox***  ***[told_cause] = ‘1’***  ***required*** | 1, Atrial fibrillation (AF, AFib)  ***Atrial Fibrillation (also called AFib or AF) is a quivering or irregular heartbeat (arrhythmia) that can lead to blood clots, stroke, heart failure and other heart-related complications. Some people refer to AF as a quivering heart***  2, Atrial flutter (flutter)  ***A particular fast heart rhythm originating in the atrium (top chamber) that results in a rapid heart rhythm. The ECG has a particular appearance that makes it able to be diagnosed by your physician. Sometimes atrial flutter is treated with medications, blood thinners, can be converted by a shock or an ablation. While it is related to atrial fibrillation, it is a distinct entity.***  3, Supraventricular tachycardia (SVT)  ***A generic term for a group of fast heart rhythm involving the top chambers (atria). This is distinct from ventricular tachycardia.***  4, Wolff-Parkinson-White (WPW)  ***A particular kind of supraventricular tachycardia (fast heart rhythm) caused by an extra electrical connection between the atria and ventricle (called a bypass tract). Sometimes this can be picked on ECG as an abnormality called a delta wave or pre-excitation.***  5, AV Nodal Reentrant Tachycardia (AVNRT)  ***A particular kind of supraventricular tachycardia (fast heart rhythm) where the ‘short-circuit’ is located in the AV node (the electrical connection between the top and bottom chambers of the heart).***  6, Atrial tachycardia or PAT  ***A particular kind of supraventricular tachycardia (fast heart rhythm) where the rapid firing spot is located in the top chamber (atria).***  7, Ventricular tachycardia (VT)  ***A particular kind of fast heart rhythm originating in the bottom chambers (ventricles).***  8, PACs or PVCs (premature beats)  ***A PAC is an extra beat originating from the top chamber (atria). A PVC is an extra beat originating from the lower chamber (ventricle). The extra beat is often followed by a pause, which sometimes is the only thing felt. These are typically diagnosed on ECG or Holter monitors.***  9, Bradycardia (slow heart rates)  10, Other  11, Still being evaluated by my doctor  12, Don’t know |
| 1-09d Fainting Survey | |
| What treatment have you received for your fainting (loss of consciousness)? Check all that apply.  ***treatment_options***  **checkbox**  ***[treatment] = ‘1’***  ***Not required*** | 1, Seizure medications  2, Medications (other than for seizures)  3, Ablation  ***An ablation is a catheter procedure done in the EP lab through your veins to ‘burn’ or ‘freeze’ to correct abnormal electrical areas in your heart.***  4, Pacemaker  ***Pacemakers are implanted devices that monitor and regulate the rhythm of the heart and send electrical signals to stimulate the heart if it's beating too slowly (bradycardia).***  5, Implantable Cardioverter - Defibrillator (ICD)  ***Implanted Cardioverter-Defibrillators (ICDs) are implanted devices that continuously monitor the heart rhythm, automatically function as pacemakers for heart rates that are too slow, and deliver life-saving shocks if a dangerously fast heart rhythm is detected, such as ventricular tachycardia (VT) or ventricular fibrillation (VF), the major cause of cardiac arrest. ICDs are generally implanted to treat or prevent life-threatening fast heart rhythms (VT or VF). All of these also have pacemaker capabilities, whereas a regular pacemaker can't deliver a high-energy shock.***  6, Don’t know |
| 1-05d General Family History Survey | |
| Does anyone in your immediate or extended biological family have, or have had, an ICD (implantable cardioverter-defibrillator), that you know of?  ***Implanted Cardioverter-Defibrillators***  ***Implanted Cardioverter-Defibrillators (ICDs) are implanted devices that continuously monitor the heart rhythm, automatically function as pacemakers for heart rates that are too slow, and deliver life-saving shocks if a dangerously fast heart rhythm is detected, such as ventricular tachycardia (VT) or ventricular fibrillation (VF), the major cause of cardiac arrest. ICDs are generally implanted to treat or prevent life-threatening fast heart rhythms (VT or VF). All of these also have pacemaker capabilities, whereas a regular pacemaker can't deliver a high-energy shock.***  ***icd***  ***radio***  ***none***  ***required*** | 1, Yes  2, No  3, Don’t know |
| 1-05a/b/c Family History – Mother/Father/Siblings | |
| A cardiac arrest?  ***Cardiac Arrest***  ***Cardiac Arrest (or sudden cardiac arrest) occurs when the heart abruptly stops beating for long enough to result in collapse and the need for CPR or an electrical shock in order to avoid immediate death. This can happen either because the electrical signals of the heart stop completely or are too fast and irregular (ventricular fibrillation). If this occurs, blood stops flowing to the brain and other vital organs.***  ***mother_card***  ***father_card***  ***brother_card***  ***sister_card***  ***radio***  ***none***  ***required*** | 1, Yes  2, No  3, Don’t know |

Supplementary Figure 3: Five year follow up survey sent through REDCap to all enrolled MVP positive respondents.


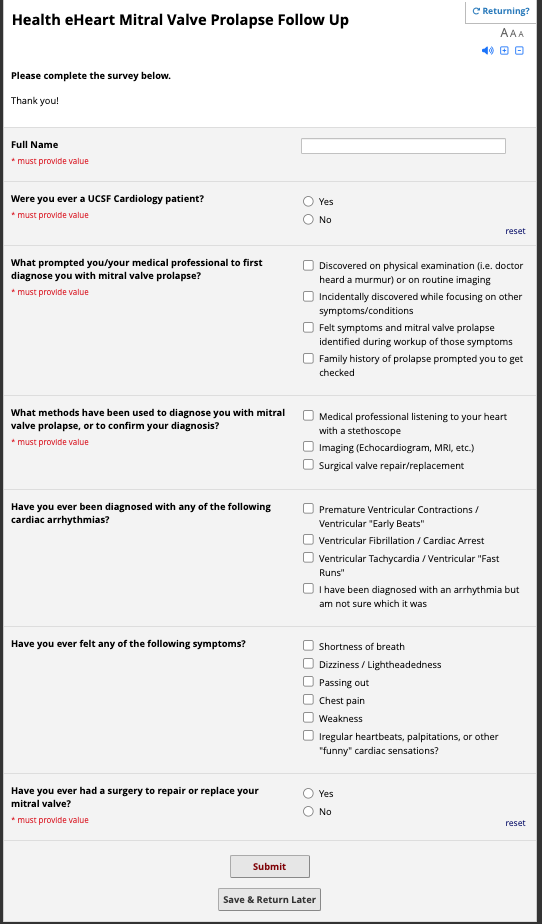

Supplement: Multimedia Appendix 1 [file formative-v10-e77968-s001.docx]
